# Organization of psychological rehabilitation and medical care for patients with mental health issues caused by military operations

**DOI:** 10.1192/j.eurpsy.2023.2083

**Published:** 2023-07-19

**Authors:** M. Arakelyan, T. Tunyan, K. Grigoryan

**Affiliations:** 1 Yerevan State Medical University after Mkhitar Heratsi; 2 The Ministry of Defence, RA, General Staff; 3 Kanaz Military Hospital, Psychiatry, Yerevan, Armenia

## Abstract

**Introduction:**

On 11 October, 2020, a post-traumatic psychological rehabilitation (PPR) department was formed in the Armed Forces owing to the last Nagorno-Karabakh war on 27 September 2020. Initially, it was located in one of the military training centers, and a day after the end of the war, on November 10, it was moved to the “Mountain Armenia” rest house in Dilijan to ensure the continuous process of rehabilitation of wounded servicemen. In addition to the qualified professional help, the favorable climatic, high-quality social-living conditions were added, which, in terms of treatment, contributes more to the transformation of the psycho-traumatic memory of the combat situation and conditions.

**Objectives:**

The objective was to sort military personnel who suffered as a result of hostilities, psychological, post-stress, mental illnesses that have not yet been diagnosed, inpatient treatment, and psychological support.

The composition of patients treated in the PPR center by category.

Compulsory military service, officers and NCOs, contract servicemen, conscripted by mobilization.

All the patients admitted with acute stress reactions or PTSD also had the following conditions:

- Mine debris (after processing),
- Gunshot wound /after treatment/,
- Closed Head Injury,
- Witnessed the death of a comrade-in-arms or close brother or father or childhood friend,
- Provided assistance to a wounded or mutilated corpse,
- They were under siege, in captivity.

**Methods:**

The following therapeutic methods were used in the Center.

Pharmacotherapy

- Antidepressants, tranquilizers, herbal preparations, nootropics, atypical neuroleptics, symptomatic medication.

Group and individual psychotherapy and psychodiagnostics

- CBT/REBT, EMDR, Biofeedback, Art Therapy, Music Therapy, Sport Activity etc.

**Results:**

From 11 October, 2020 to 24 December, 2021, more than 700 military personnel received inpatient treatment in the post-traumatic rehabilitation unit, the vast majority of whom were discharged from the unit with improvement to continue their service.

**Conclusions:**

In the post-war period, the number of suicides and suicidal attempts has noticeably increased in society. None of the military personnel who received treatment through the specialized activities of the PPR Center and returned to further military service committed suicide or attempted suicide over the entire subsequent service.

**Acknowledgement:**

“The Mountain Armenia” Psychological Rehabilitation Center was carried out its work by support of the Franco-Swiss international organization «Santé Arménie», which conducted international professional supervision for a year and financed the work of psychologists working part-time at the Center. We would like to express our deepest gratitude to the «Santé Arménie» which support was invaluable.

**Disclosure of Interest:**

None Declared

